# A Mechanism of Gene Amplification Driven by Small DNA Fragments

**DOI:** 10.1371/journal.pgen.1003119

**Published:** 2012-12-13

**Authors:** Kuntal Mukherjee, Francesca Storici

**Affiliations:** School of Biology, Georgia Institute of Technology, Atlanta, Georgia, United States of America; Duke University, United States of America

## Abstract

DNA amplification is a molecular process that increases the copy number of a chromosomal tract and often causes elevated expression of the amplified gene(s). Although gene amplification is frequently observed in cancer and other degenerative disorders, the molecular mechanisms involved in the process of DNA copy number increase remain largely unknown. We hypothesized that small DNA fragments could be the trigger of DNA amplification events. Following our findings that small fragments of DNA in the form of DNA oligonucleotides can be highly recombinogenic, we have developed a system in the yeast *Saccharomyces cerevisiae* to capture events of chromosomal DNA amplification initiated by small DNA fragments. Here we demonstrate that small DNAs can amplify a chromosomal region, generating either tandem duplications or acentric extrachromosomal DNA circles. Small fragment-driven DNA amplification (SFDA) occurs with a frequency that increases with the length of homology between the small DNAs and the target chromosomal regions. SFDA events are triggered even by small single-stranded molecules with as little as 20-nt homology with the genomic target. A double-strand break (DSB) external to the chromosomal amplicon region stimulates the amplification event up to a factor of 20 and favors formation of extrachromosomal circles. SFDA is dependent on Rad52 and Rad59, partially dependent on Rad1, Rad10, and Pol32, and independent of Rad51, suggesting a single-strand annealing mechanism. Our results reveal a novel molecular model for gene amplification, in which small DNA fragments drive DNA amplification and define the boundaries of the amplicon region. As DNA fragments are frequently found both inside cells and in the extracellular environment, such as the serum of patients with cancer or other degenerative disorders, we propose that SFDA may be a common mechanism for DNA amplification in cancer cells, as well as a more general cause of DNA copy number variation in nature.

## Author Summary

DNA amplification is a copy-number increase of a DNA segment. Although DNA amplification is frequently observed in cancer and other degenerative disorders, the molecular mechanisms initiating this process are still largely elusive. Here we demonstrate that small DNA fragments with homology to two distant loci on the same chromosomal arm can trigger amplification of the region between the loci in yeast *S. cerevisiae.* Small fragment-driven DNA amplification (SFDA) is detected as intrachromosomal tandem duplications or extrachromosomal circles. Furthermore, a double-strand break several kilobases from the chromosomal amplicon region stimulates SFDA. SFDA efficiency depends on the homology length shared by the small DNAs and the target chromosomal loci. Homology as short as 20 nucleotides and even single-stranded molecules trigger SFDA. These results reveal a novel mechanism for initiating gene amplification, which could occur in cancer cells and could contribute to copy-number polymorphisms driving genetic variation in humans and other organisms.

## Introduction

DNA amplification is defined as a molecular process resulting in copy number increase of a discrete chromosomal DNA region. DNA amplification is found in many tumors, it is associated with several neuropathies and it can affect the susceptibility to certain diseases, such as systemic lupus erythematosus [1], [2]. It is believed that DNA copy number increase is a major molecular mechanism driving oncogenesis in many kinds of cancer, and it affects tumor progression and clinical outcome [3]. Gene amplification can in fact minimize the efficacy of drugs via overproduction of a protein that may be a drug target or via overproduction of a factor, which inactivates or eliminates the drug [4], [5]. DNA amplification, together with DNA copy number reduction is a major source of genetic variation, which is not necessarily always pathogenic, but which can lead to polymorphisms between individual genomes in humans and other organisms [6]–[14].

Amplified DNA is commonly detected cytogenetically as repeated units clustered at a single chromosomal locus (homogeneously staining region, HSR) or as circular extrachromosomal elements replicating autonomously and lacking a centromere and telomeres, termed double minutes (DMs) [15], [16]. DMs segregate randomly during mitosis and are therefore very unstable, except when they provide a selective advantage to the cells by carrying extra copies of oncogenes or drug resistance genes [17].

In current models of DNA amplification a double-strand break (DSB) is regarded as the principal initiator [18]–[20]. The breakage-fusion-bridge (BFB) cycle proposed by Barbara McClintock is a well-established mechanism of gene amplification also found in tumors [21], [22]. BFB involves the repeated breakage and fusion of isochromatids following the loss of a telomere, resulting in inverted duplications. Palindromic sequences, which are hot spots for DSBs, are in fact a major source of chromosomal rearrangements and gene amplification with formation of inverted duplications [23]–[27]. In addition, a DSB can trigger amplification events by promoting non-allelic recombination between sequences containing direct repeats [28]. Finally, break-induced replication (BIR) can lead to duplications, when broken DNA uses ectopic homology to start replication fork, as well as to double rolling-circle replication events [29].

Mechanisms for gene amplification that do not necessitate a DSB include various processes of template switching. The model of replication fork stalling and template switching (FoSTeS) has been suggested to explain the complex duplication and deletion rearrangements associated with Pelizaeus-Merzbacher disease and potentially other non-recurrent rearrangements of the human genome [30], [31].

Comparative genomic and structural studies of extrachromosomal DNA elements have revealed that the initial separation of DMs and episomes from their original genomic locus may often occur by mechanisms that do not leave a scar or any alteration at the chromosomal region carrying the original amplicon DNA [32]. DNA excision following loop formation or postreplicative excision of a chromosomal fragment has been suggested to explain the amplification of the MYCN oncogene and the epidermal growth factor receptor gene (EGFR), respectively [32], [33]. As in the case of the EGFR, there are many other examples of gene amplification in cancer cells in which the original amplicon region is retained intact at its normal locus in the genome and the initial cause of the amplification event is obscure [32], [34]. Hence, despite the current knowledge on the mechanisms of gene amplification, still much remains unknown about the molecular triggers that induce DNA amplification and define the boundaries of the amplicons, especially when the initiating process does not depend on a DSB in the chromosomal region carrying the amplicon.

In previous work in yeast, we showed that small DNA molecules in the form of synthetic oligonucleotides (oligos) are potent tools for genome engineering and rearrangements, as oligos can drive chromosomal point mutations, deletions, fusions, or translocations both in the presence and in the absence of a DSB in the targeted DNA [35]–[37]. In the current study, exploiting the use of oligos, we investigated whether small DNA fragments, even as single-stranded molecules can trigger events of gene amplification and we developed an approach to capture such events in *Saccharomyces cerevisiae*. Our results uncover a novel pathway in which small DNA molecules are drivers of DNA amplification, small fragment-driven DNA amplification (SFDA), and we provide initial characterization of its molecular mechanisms.

## Results

### Experimental system to capture SFDA events

**Figure 1:**
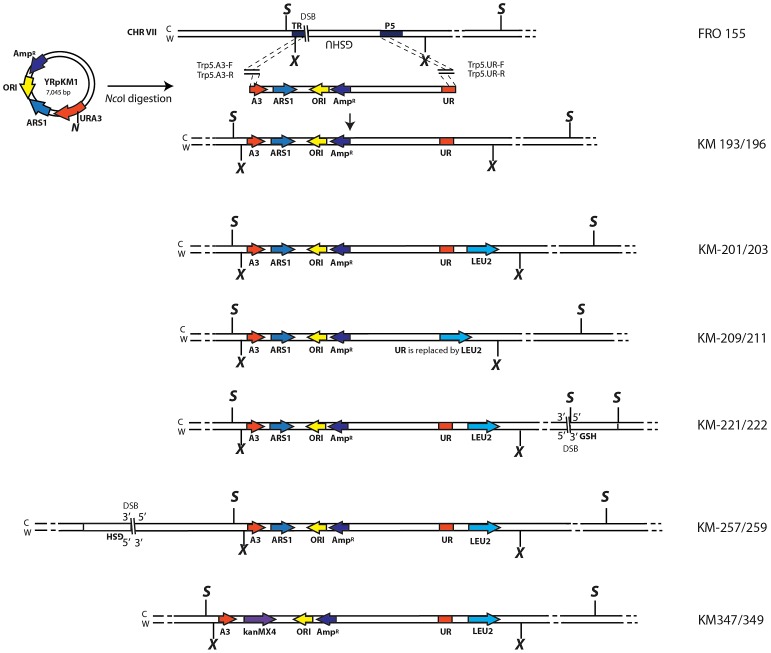
Schemes of different strains containing the A3-UR amplicon cassette in chromosome VII. The yeast strain FRO-155 has a GSHU cassette in the middle of the *TRP5* gene on chromosome VII. (C) and (W) indicate the Crick and Watson strands. The yeast/*E. coli* shuttle plasmid YRpKM1, containing the *Amp^R^* gene (dark blue), *ORI* (yellow), the yeast *ARS1* (blue) and the yeast *URA3* marker gene (red), was used to generate the amplicon cassette. In order to integrate the amplicon cassette into yeast chromosomal DNA, YRpKM1 was linearized by the *Nco*I (N) enzyme in the middle of the *URA3* gene. The linearized YRpKM1, A3-UR cassette, was integrated into *S. cerevisiae* chromosome VII at the site of an I-*Sce*I DSB within the *TRP5* locus of strain FRO-155 following co-transformation with two pairs of oligos (Trp5.A3.F, Trp5.A3.R/Trp5.UR.F, Trp5.UR.R, see Table S1) complementary to the ends of the A3-UR cassette and to the *TRP5* broken ends, according to the gene collage protocol, generating strains KM193 and KM196. Correct integration of the A3-UR amplicon cassette was confirmed by PCR and sequence analysis. Yeast strains KM-201,203 and KM-209,211 derive from KM-193 and KM-196, respectively. KM-201,203 strain contain a *LEU2* marker (light blue) integrated downstream of the UR sequence. In strain KM-209,211 the UR sequence is replaced by the *LEU2* gene. The KM-221,222 and KM-257,259 strains are variant forms of KM-201,203 containing the GSH cassette with the I-*Sce*I endonuclease gene under the inducible *GAL1* promoter, the hygromycin resistance marker and the I-*Sce*I cutting site, which was integrated 10 kb downstream or upstream from the A3-UR amplicon cassette, respectively. The KM-347,349 strains are variant forms of KM201/203 in which *ARS1* has been replaced with *kanMX4*. The *Xba*I (X) and the *Sac*I (S) restriction sites present on the chromosomal tracts without and with the amplicon are indicated.

**Figure 2:**
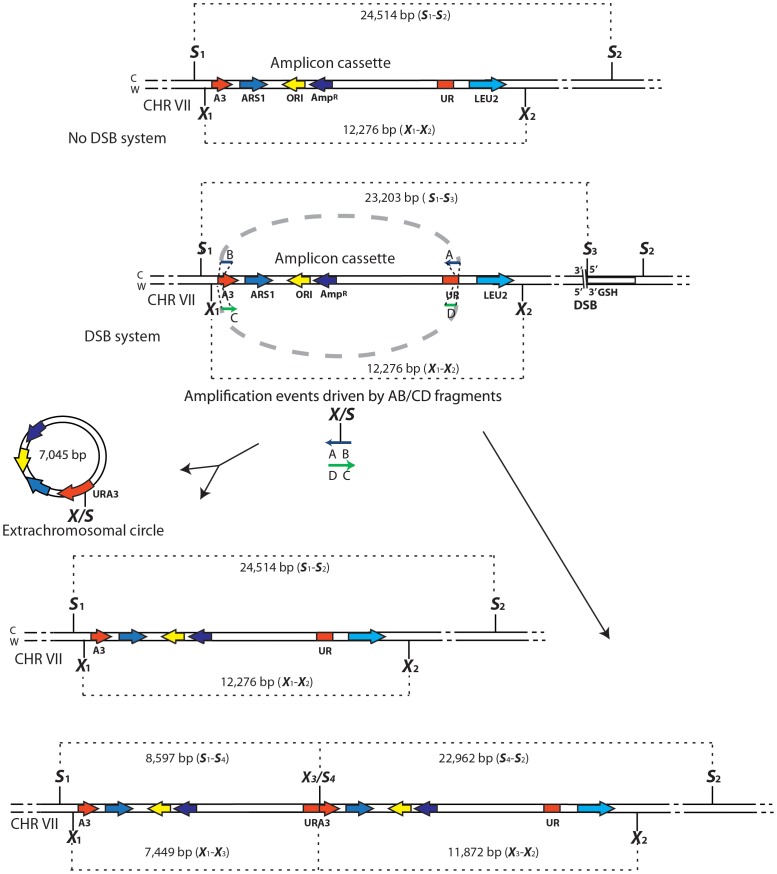
Diagrams of SFDA products. Schemes of the region containing the A3-UR amplicon cassette on chromosome VII in strains KM201/203 (No DSB system) and KM221/222 (DSB system) are shown. The AB (in blue) and CD (in green) oligos (not to scale), used to initiate the SFDA events, are complementary to each other and complementary to the C or W strand, respectively. The thick dotted grey line connecting the AB or CD oligo halves indicate that the two halves of the oligos are not separated from each other. The *Sac*I (S) and *Xba*I (X) restriction enzyme sites present on the DNA molecules containing the amplicon and on the AB and CD oligos are indicated. If more than one site for *Sac*I or *Xba*I is present on the same molecule of the amplicon these sites are identified by a small number. The base-pair sizes of all the *Sac*I or *Xba*I digestion products of the regions containing the amplicon cassette are shown. The products of SFDA as intact chromosomal amplicon plus extrachromosomal circle or as tandem duplication of the amplicon cassette on the chromosome are shown.

We have hypothesized that small DNA fragments with complementarity to chromosomal DNA can be the initiators of DNA amplification (SFDA) events. In order to capture such events, we have developed a procedure based on the well-known plasmid gap-repair assay in yeast [38]. In the gap-repair assay, yeast cells are transformed with a plasmid containing a gap within a region that has homology to yeast chromosomal DNA, and the gap is repaired by gene conversion from the chromosomal locus. The plasmid remains extrachromosomal if gene conversion occurs without a crossing over but the entire plasmid integrates if crossing over takes place. In our system, the gapped plasmid is reduced to 80 bp and consists of two complementary single-stranded oligos, termed AB and CD, which have homology to the sequence in the middle of the yeast *URA3* marker gene (Table S1). The DNA sequence to be amplified by the AB and CD oligos, termed the amplicon cassette, derives from an integrated copy of the replicative plasmid YRpKM1. YRpKM1was linearized at the *Nco*I site in the middle of the *URA3* marker gene and integrated by gene collage [37] into yeast chromosome VII, generating strains KM-193,196 (Figure 1 and Table S2). The yeast strains containing the amplicon cassette with the split *URA3* marker (A3-UR cassette) cannot grow on medium lacking uracil (Ura^−^). Because the AB and CD oligos have homology to each half of the split *URA3* marker, we hypothesized that recombination between the oligos and the amplicon cassette, as in a gap-repair event, could restore a functional *URA3* marker resulting in formation of either an extrachromosomal circle or a duplication of the amplicon region (Figure 2). Therefore, appearance of yeast colonies with the Ura^+^ phenotype would be indicative of SFDA events driven by the AB and CD oligos.

### SFDA occurs efficiently and it can be driven by short double- or single-stranded DNAs

In order to ensure that the reconstituted *URA3* gene can only form in our system following oligo-driven amplification of the amplicon region containing the split A3-UR sequence and not by oligo-driven recombination with residual YRpKM1 DNA, which might have been randomly integrated in the KM-193,196 strains, these strains were modified to generate KM-201,203 and KM-209,211 (Figure 1). In the KM-201,203 strains the *LEU2* marker was inserted immediately downstream from the UR segment (Figure 1 and Figure 2), while in the KM-209,211 strains the *LEU2* marker replaced completely the UR sequence (Figure 1). We expected formation of Ura^+^ colonies following transformation with the AB and CD oligos only in strains KM-201,203 but not in KM-209,211 if there were no other *URA3* sequence present in these cells. Moreover, the AB and CD oligos were designed to introduce either a *Sac*I (AB_S_ and CD_S_) or an *Xba*I (AB_X_ and CD_X_) restriction site (Table S1) in the middle of the reconstituted *URA3* gene. Thus, oligo-driven amplification of the A3-UR amplicon cassette can be confirmed by PCR of the reconstituted *URA3* gene and subsequent restriction digestion of the PCR product by either the *Sac*I or the *Xba*I enzyme.

**Figure 3:**
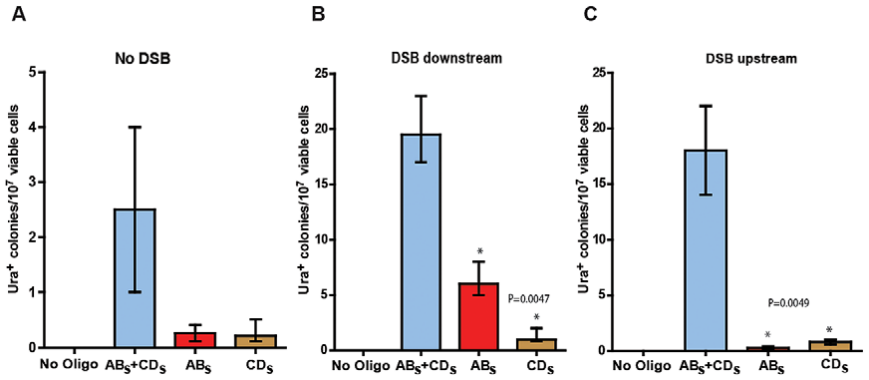
SFDA efficiency without and with a DSB. Presented are numbers of Ura^+^ colonies per 10^7^ viable cells obtained after transformation of yeast cells with no oligos, AB_S_ and/or CD_S_ oligos. The vertical bars correspond to the median values from six determinations; the error bars represent the range. (A) Strains KM-201,203; (B) strains KM-221,222, in which a DSB was induced 10 kb downstream from the amplicon cassette prior to oligo transformation; and (C) strains KM-257,259, in which a DSB was induced 10 kb upstream of the amplicon cassette prior to oligo transformation. Frequency values obtained for the single-stranded AB_S_ and CD_S_ oligos in a given strain background were compared with each other by the Mann-Whitney test and the *p* values of the significant differences, highlighted by the asterisks, are given on top of the corresponding bars.

With the goal of testing the capacity of small DNA fragments to drive amplification of chromosomal regions in yeast, KM-201,203 and KM-209,211 cells were transformed with the AB_S_ and CD_S_ or with the AB_X_ and CD_X_ oligos. While transformation of KM-209,211 strains, which contain only the A3 sequence but not the UR, yielded no Ura^+^ transformants out of more than 10^9^ cells, KM-201,203 cells produced Ura^+^ colonies with a frequency of ∼2/10^7^ viable cells (Figure 3A and Figure S1A). Four out of four random Ura^+^ colonies derived from KM-201,203 strains that were analyzed by colony PCR and *Xba*I or *Sac*I restriction digestion revealed the presence of the *Sac*I or *Xba*I site in the reconstituted *URA3* gene, respectively (not shown). Transformation of KM-201,203 by either only AB_S_, CD_S_, AB_X_ or CD_X_ single-stranded DNAs also resulted in Ura^+^ colonies and the single-stranded oligos were about 10-fold less effective than the mix of complementary oligos (Figure 3A and Figure S1A). No significant strand bias was revealed between AB_S_ and CD_S_ or AB_X_ and CD_X_ molecules.

### A DSB proximal to the amplicon region stimulates SFDA

We then investigated if a DSB occurring outside of the amplicon region, but in its vicinity either downstream or upstream would affect SFDA frequency. We inserted the site for the I-*Sce*I site-specific nuclease and the I-*Sce*I gene regulated by the galactose inducible promoter ∼10 kb downstream or upstream of the amplicon cassette in strain KM-201,203 generating strains KM-221,222 and KM-257,259 (Figure 1 and Figure 2, and Table S2). The induction of the DSB to the side of the amplicon cassette increased SFDA by the AB_S_ and CD_S_ or the AB_X_ and CD_X_ oligos 6–9-fold (Figure 3B and Figure S1). Break induction led to a large increase in SFDA also driven by single-stranded oligos, up to 20-fold, but with a strong strand bias in favor of the oligos that were complementary to the broken chromosomal 3′ end. These were the AB_S_ and the AB_X_ oligos when the break was induced downstream of the amplicon in KM-221,222, and the CD_S_ and the CD_X_ oligos when the DSB was induced upstream of the amplicon in KM-257,259 (Figure 3B, 3C and Figure S1B, S1C). While the strand bias reveals clear differences in the frequency of SFDA driven by the AB_S/X_ and CD_S/X_ oligos to either side of the DSB, the absolute SFDA frequencies for the AB_S/X_ and CD_S/X_ oligos are much higher when the DSB is induced downstream of the oligo targeting region than upstream of it. Such differences in oligo recombination frequency following a DSB induced to either the side of the targeting region were also previously found [39] and may be dependent on the sequence context or on unequal resection efficiency at the DSB ends.

The AB_S/X_ and CD_S/X_ 80-mers used in the experiments described above, have homologous sequences of 40 nt to either side of the split A3-UR marker and this homology was sufficient to stimulate SFDA both in the presence and in the absence of a DSB outside the amplicon region. To determine if DNA fragments with asymmetric homology distribution to the amplicon region could drive SFDA, we utilized A20B60_S_ and C60D20_S_ 80-mers having 20 bases of homology to one side of the amplicon and 60 bases of homology to the other side of the amplicon (Table S1). Following DSB induction downstream of the amplicon region in strains KM-221,222, and without the induction of a DSB in strains KM-201,203, we detected rare SFDA events by these oligos transformed as pairs, as well as single strands (Table S3). These data suggest that DNA fragments sharing even very short tracts of homology with genomic DNA can trigger SFDA of a chromosomal segment.

### SFDA results in formation of extrachromosomal circles and/or duplications

**Figure 4:**
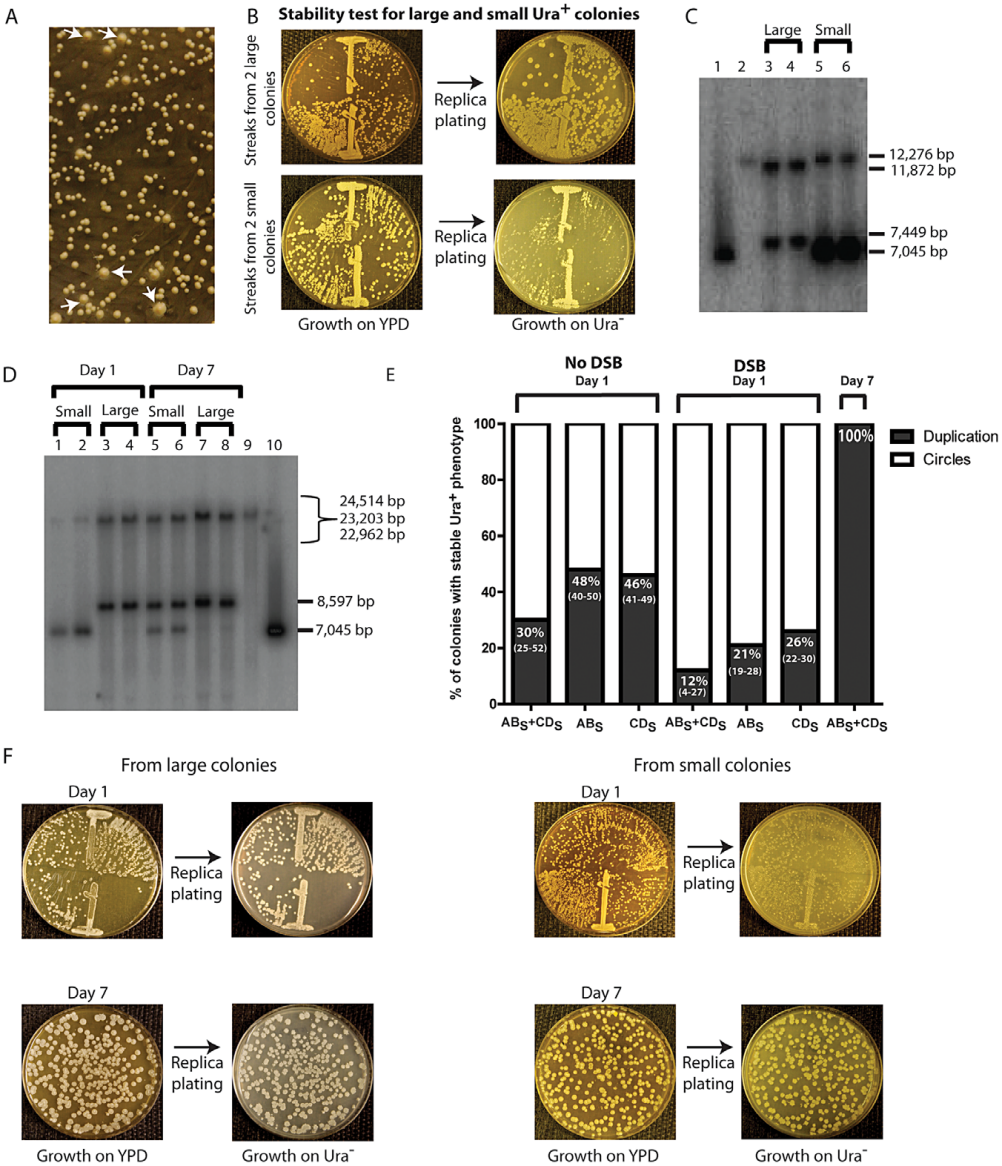
Phenotypic and molecular characterization of SFDA. (A) Ura^+^ colonies from KM-221 on Ura^−^ medium after DSB induction and transformation by AB_S_+CD_S_. Arrows point to large colonies. (B) Ura^+^-stability test for two large and two small Ura^+^ colonies from strain KM-221 after DSB induction and transformation by the AB_S_+CD_S_ oligos. (C) Detection of the amplicon region from large and small Ura^+^ colonies derived after DSB induction and transformation by the AB_X_ and CD_X_ oligos. Lane 1, control YRpKM1 linearized (7,045-bp); lane 2, control genomic DNA from KM-221 Ura^−^ cells (12,276-bp); lanes 3, 4 genomic DNA from large Ura^+^ KM-221 colonies (7,449-bp, 11,872-bp); lanes 5, 6 genomic DNA from small Ura^+^ KM-221 colonies (7,045-bp, 12,276-bp). (D) Detection of the amplicon region from large and small Ura^+^ colonies derived after DSB induction and transformation by the AB_S_ and CD_S_ oligos. Lanes 1, 2, genomic DNA from small Ura^+^ KM-221 colonies grown 1 day in Ura^−^ medium (7,045-bp, 24,514-bp); lanes 3, 4, genomic DNA from large Ura^+^ KM-221 colonies grown 1 day in Ura^−^ medium (8,597-bp, 22,962-bp); lanes 5, 6, genomic DNA from small Ura^+^ KM-221 colonies grown 7 days in Ura^−^ medium (7,045-bp, 8,597-bp, 22,962-bp); lanes 7, 8, genomic DNA from large Ura^+^ KM-221 colonies grown 7 days in Ura^−^ medium (8,597-bp, 22,962-bp); lane 9, control genomic DNA from KM-221 Ura^−^ cells (23,203-bp); lane 10, control YRpKM1 linearized (7,045-bp). (E) Percentage of colonies with stable Ura^+^ phenotype (indicative of duplication) following transformation by AB_S_ and/or CD_S_ oligos without DSB and with DSB at day 1 and day 7 (median and range). (F) Ura^+^-stability test for the large and small Ura^+^ colonies analyzed in (D). Single-colony streaks or dilutions on YPD and results of replica plating on Ura^−^ medium for colonies taken at day 1 or 7 of growth in the Ura^−^ medium are shown.

We observed that among the yeast colonies forming on the Ura^−^ medium following transformation by the AB_S/X_ and/or CD_S/X_ oligos, either in the presence or in the absence of a DSB, some colonies were clearly larger in size than others (Figure 4A). A large colony size is indicative of a stable Ura^+^ phenotype, while a small colony size is indicative of an unstable Ura^+^ phenotype. In order to confirm this in cells transformed by the AB_S/X_ and CD_S/X_ oligos, we streaked 20 large and 20 small size colonies both from KM-221 and KM-222 on rich YPD medium and after two days of growth, replica-plated them to Ura^−^ medium. All 40 large-colony streaks showed a stable Ura^+^ phenotype, while all 40 small-colony streaks showed an unstable Ura^+^ phenotype (see Figure 4B).

A stable Ura^+^ phenotype suggests chromosomal integration of *URA3*, such as that formed from duplication of the amplicon cassette. An unstable Ura^+^ phenotype would imply that the *URA3* gene is carried on an unstable molecule, such as that formed from circularization of the amplicon cassette, which derives from the YRpKM1 plasmid that does not have a centromere and is frequently lost. YRpKM1 is present in only 9% (median value of six repeats and range of 7% to 10%) of cells maintained on Ura^−^ medium. We therefore examined whether the large and small colonies contained a duplication of the amplicon cassette or extrachromosomal circles with the *URA3* gene, respectively. We extracted genomic DNA from two large and two small Ura^+^ colonies following transformation with the AB_X_ and CD_X_ and from two large and two small colonies derived by transformation with the AB_S_ and CD_S_ oligos. A Southern blot of genomic DNA digested with either *Xba*I or *Sac*I was probed with part of the ampicillin resistance marker (see Materials and Methods). The resulting band pattern proved that SFDA corresponded to either a tandem duplication of the amplicon in stable Ura^+^ colonies or to formation of extrachromosomal amplicon circles in unstable Ura^+^ colonies (Figure 4C in lanes 3–6 and Figure 4D in lanes 1–4, and Figure 2). Moreover, in Figure 4C (lanes 5 and 6) and 4D (lanes 1 and 2) it is evident that the band corresponding to linearized circles is substantially more intense than the band corresponding to the intact chromosomal amplicon region in each lane, suggesting that there are several copies of extrachromosomal circular DNA molecules. The amplified amplicon cassette, in addition of having a functional *URA3* gene and an autonomous replicating sequence (*ARS1*), also contains an origin for replication and a selectable marker for *E. coli* cells. Thus, if the amplicon is in the form of an extrachromosomal circle, this can be rescued into *E. coli* cells. In six small colonies (2 deriving from KM-201, 2 from KM-221 and 2 from KM-222 transformed by AB_X_+CD_X_ or AB_S_+CD_S_) examined, we were able to rescue extrachromosomal circles of the amplicon cassette in *E. coli*. These plasmids had the expected restriction pattern of the circular amplicon and also contained either the *Xba*I or *Sac*I restriction site introduced by the AB_X/S_ or CD_X/S_ oligos, respectively (Figure S2).

The percentage of colonies with a stable Ura^+^ phenotype was higher in the no DSB system than in the DSB system (*p* = 0.0006 for AB_S_ and CD_S_ oligos; *p* = 0.009 for AB_S_ oligo; *p* = 0.009 for CD_S_ oligo), and was higher for cells transformed with single-stranded oligos than with oligo pairs in both systems (in the no DSB system *p* = 0.0273 for AB_S_ compared with AB_S_ and CD_S,_, *p* = 0.0208 for CD_S_ compared with AB_S_ and CD_S_; in the DSB system *p* = 0.0156 for AB_S_ compared with AB_S_ and CD_S_, *p* = 0.0032 for CD_S_ compared with AB_S_ and CD_S_) (Figure 4E).

### Persistent growth in selective medium allows for secondary events of chromosomal amplicon duplication

We next examined if prolonged growth in selective medium for cells containing the extrachromosomal circles could promote integration of the circles, resulting in duplication of the entire region. One small and one large colony from KM-221 and KM-222 were examined for the stability of their Ura^+^ phenotype and the molecular configuration of their amplicon region after one and seven days of growth in the Ura^−^ selective medium (see Materials and Methods). Results shown in Figure 4D (lanes 5–8) and Figure 4F revealed that a stable Ura^+^ phenotype corresponding to tandem duplication of the amplicon are observed following persistent growth in selective Ura^−^ medium also of cells initially derived from small colonies. The corresponding day-7 genomic extracts of cells showed both a band corresponding to the linearized circles and one corresponding to the tandem duplication (Figure 4D, lanes 5 and 6).

### SFDA that generates extrachromosomal circles, but not duplications, requires the presence of an ARS in the amplicon

**Figure 5:**
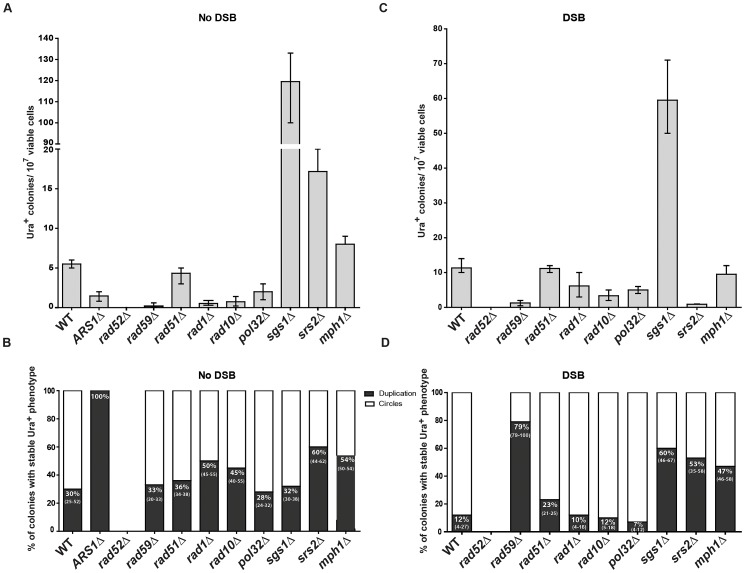
Genetic requirements for SFDA. Presented are numbers of Ura^+^ colonies per 10^7^ viable cells obtained after transformation by AB_S_ and CD_S_ oligos. The vertical bars correspond to the median values from at least six independent transformations; the error bars represent the range. (A) Strains used were KM-201,203 and derivative mutants. (C) Strains used were KM-221,222 and derivative mutants. (B) and (D) Percentage (median and range) of colonies with stable Ura^+^ phenotype (indicative of duplication) within four samples of 45 Ura^+^ colonies for each strain from the experiment shown in (A) or (C), respectively. For the percentage data shown in (B) for *rad59*Δ three groups of 12, 13, 12 Ura^+^ colonies and in (D) for *sgs1*Δ eight groups of 45 Ura^+^ colonies and for *rad59*Δ three groups of 33, 35, 68 colonies were screened. For the *ARS1*Δ strains all groups had 100% stable Ura^+^ colonies.

To determine if and how the presence of an ARS sequence in the amplicon region affects SFDA, we deleted the *ARS1* present in the amplicon region in strains KM-201,203 and transformed the new strains (KM-347,349) with the AB_S_ and/or CD_S_ oligos. As presented in Figure 5A and Figure S3A, we found that SFDA can also occur at DNA regions that do not contain an ARS element. However, in the absence of the ARS sequence, all SFDA events that can be detected are duplications, as the extrachromosomal circles that might form cannot be maintained if devoid of an ARS (Figure 5B and Figure S3B). In the strains in which we deleted the *ARS1* sequence of the amplicon, the frequency of colonies with a stable Ura^+^ phenotype did not change when both AB_S_ and CD_S_ oligos were used (*p* = 0.5228), while it was reduced 2–3-fold when individual single-stranded oligos were used (*p* = 0.0090 for AB_S_ oligo, *p* = 0.0043 for CD_S_ oligo).

### SFDA is dependent on *RAD52, RAD59*, *RAD1*, *RAD10*, and *POL32*, but not on *RAD51*

In order to identify the molecular processes driving SFDA, we investigated the genetic requirements that are at the basis of this DNA amplification mechanism. We deleted *RAD52*, which is essential for recombination both via single-strand annealing (SSA) and via strand invasion [40], and it is also implicated in end joining between DSBs with complementary single-strand ends [41]. SFDA was completely dependent on Rad52 function both in the absence and in the presence of an induced DSB (Figure 5A and 5C). The deletion of *RAD59*, a *RAD52* homolog, also greatly reduced SFDA, and SFDA frequency partially decreased (2 to 8 fold) in *rad1* (*p* = 0.0045) and *rad10* (*p* = 0.0045) mutants without the DSB, as well as with the DSB (*p* = 0.0159 for *rad1* and *p* = 0.0047 for *rad10*) (Figure 5A and 5C). There was, however, no effect of loss of Rad51, which is the recombinase that mediates strand invasion of duplex DNA [40] (Figure 5A and 5C), supporting an SSA mechanism. We then examined the role of Pol32, a non-essential subunits of *S. cerevisiae* replicative DNA polymerase δ, which is uniquely required in BIR to re-establish DNA replication at stalled and broken replication forks and at chromosomes with truncated ends [42]. While the effect was much smaller than that occurring in BIR, the *pol32* deletion reduced SFDA 2–3-fold both in the presence and in the absence of an induced DSB near to the amplicon region (Figure 5A and 5C). Interestingly, SFDA events following DSB induction were dominated by formation of extrachromosomal circles in all backgrounds (*p* = 0.0006 in wild type, *p* = 0.0050 in *rad51*, *rad1*, *rad10* and *pol32*) with the exception of *rad59*. In the absence of *RAD59*, the rare SFDA events that still occurred in the break system were mostly (79%) duplications (Figure 5B and 5D).

### Role of DNA helicases in SFDA

To identify further players in SFDA, and in particular factors that may favor formation of duplications over extrachromosomal circles, we tested the requirement of the helicases Sgs1, Srs2 or Mph1, which channel recombination intermediates into noncrossover pathways [43] (and references therein). The results shown in Figure 5A revealed that deletion of each of the three helicase genes stimulates SFDA, especially that of *SGS1*, which increased the frequency of SFDA more than a factor of 20. In the presence of a DSB, the frequency of SFDA increased only in *sgs1* cells and decreased in *srs2* cells (Figure 5C). We also detected a few SFDA events by transforming *sgs1* strains with and without DSB induction using the asymmetric A20B60_S_ and C60D20_S_ oligos (Table S3). The percentage of duplications versus extrachromosomal circles was increased in all helicase mutants in the DSB system (*p* = 0.0040 for *sgs1, p* = 0.0040 for *srs2* and *p* = 0.0121 for *mph1*) and, with the exception of *sgs1*, also in the absence of a DSB (*p* = 0.8498 for *sgs1*, *p* = 0.0147 for *srs2* and *p* = 0.0396 for *mph1*) (Figure 5D and 5B).

## Discussion

In this study, we have addressed whether small DNA fragments can promote amplification of chromosomal regions several kb large. Analyses were carried out in *S. cerevisiae* cells, in which a reporter system was engineered to capture amplification events initiated by small DNA fragments. The similarity of our oligo system used to capture the SFDA events in yeast cells to the plasmid gap repair system is only structural, as the repair of plasmid double-stranded DNA gaps from either plasmid or chromosomal templates requires the function of Rad51 [44], which is instead dispensable in the SFDA mechanism. We found that small DNA fragments in the form of a pair of complementary DNA 80-mers or just single-stranded 80-mers sharing as little as 20 nt of homology with the target chromosomal DNA are capable of promoting amplifications of ∼7 kb regions in yeast. The SFDA events result in tandem chromosomal duplications or formation of extrachromosomal circles (Figure 2 and Figure 4), similar to the DNA amplification structures commonly found in many cancer cells (HSRs and DMs). While not essential for SFDA, the presence of the ARS in the amplicon region is a requirement for the detection of extrachromosomal circles (Figure 5B). These circles presumably form, but they cannot be maintained in the absence of an ARS. SFDA is clearly distinct from the breakage-fusion-bridge (BFB) cycle and other known mechanisms of DNA amplification; although a DSB stimulates SFDA, a DSB is not required to initiate SFDA, the resulting duplications are tandem rather than inverted, and the trigger is a small extrachromosomal DNA fragment. To our knowledge, this is the first demonstration that DNA fragments can be the source of a DNA duplication involving chromosomal regions.

**Figure 6:**
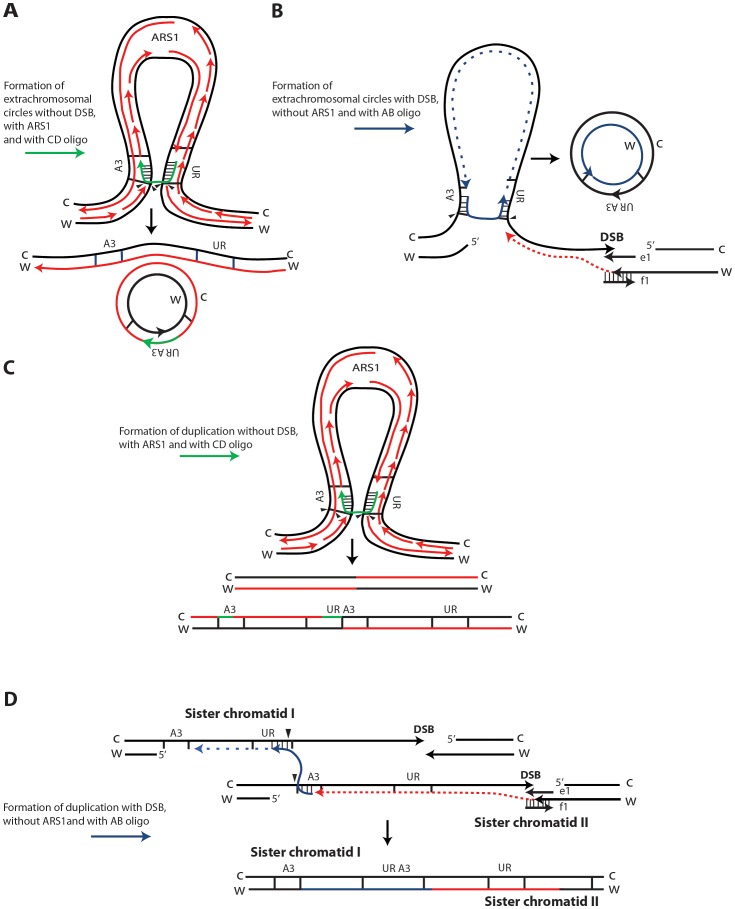
Possible SFDA mechanisms. Small DNA fragments can find homology with single-stranded sequences at the boundaries of the A3-UR amplicon region, either during DNA replication or during processing of DSB ends, and trigger amplification events resulting in formation of extrachromosomal circles or duplications. There are multiple possible mechanisms for SFDA and here we present the sketch of four SFDA events initiated by either the AB or CD oligo. Our models aim to show examples of SFDA-driven events illustrating an intermediate step for each chosen SFDA event in which the oligos are fully paired with the chromosomal sequence. (A) SFDA-driven formation of an extrachromosomal circle by the CD oligo (in green) when no DSB is induced and there is *ARS1* in the amplicon, or (B) by the AB oligo (in blue) when a DSB is induced next to the amplicon; (C) SFDA-driven formation of a tandem duplication by the CD oligo when no DSB is induced and there is *ARS1* in the amplicon, or (D) by the AB oligo when a DSB is induced next to the amplicon. DNA synthesis on the Crick (C) and Watson (W) strands is indicated by the red lines, assuming the fork comes from *ARS1* in (A) and (C). DSB repair synthesis is indicated as red dotted lines in (B) and (D). DNA synthesis primed by the AB oligo is shown as blue dotted line. The small black arrows indicate points of strand cleavage to resolve the recombination intermediate.

SFDA is Rad51 independent, but requires Rad52 and Rad59, and it is in part dependent on Rad1 and Rad10 function, indicating that the oligos anneal directly to the homologous target DNA in single-stranded regions, rather than via strand invasion [39]. As the deletion of *POL32* reduces SFDA frequency only 2 to 3-fold, while the frequency of BIR is reduced at least 20-fold in *pol32* null cells [42], we believe SFDA does not share the common mechanism with the BIR pathway. Nevertheless, the partial dependence on Pol32 suggests involvement of DNA polymerase δ in SFDA; SFDA could occur during DNA replication, or during the course of DSB repair, as both processes utilize Pol δ function [45]. As the major mechanisms of SFDA is SSA, we postulated that formation of extrachromosomal circles or duplications mainly occurs when the 3′ and 5′ ends of a single-stranded DNA fragment anneal concomitantly or sequentially with homologous single-stranded regions present on the same arm of either one or two sister chromatids (Figure 6). The higher stability of the oligo pair relative to the single-strands can be explained by a greater capacity of the pair to anneal with complementary chromosomal single-stranded regions and to engage in second-strand synthesis. Thus, in our model, the small DNA fragments, as single strands or in pairs, promote extrusion of the amplicon region from one sister chromatid, resulting in formation of an extrachromosomal circle, which in order to be maintained must segregate with an intact sister chromatid (Figure 6A, 6B). Alternatively, different resolution of the recombination intermediate, or direct annealing of a small DNA fragment to both sister chromatids promote a crossing over between sister chromatids, which results in an unequal sister chromatid exchange, generating a duplication of the amplicon region (Figure 6C and 6D, respectively).

Consistent with our model, deletion of the *MPH1*, *SRS2* or *SGS1* genes, which play a role in suppressing crossing over during recombination [43], [46], significantly (see above results) increases the percentage of SFDA duplication events over formation of extrachromosomal circles in the presence and, with the exception of *sgs1*, also in the absence of a DSB (Figure 5B, 5D). Sgs1 is a RecQ family DNA helicase and a homolog of the human BLM, WRN, and RECQL4 proteins that are mutated in Bloom's, Werner, and Rothmund Thomson syndromes, respectively. Yeast mutants defective in Sgs1 display increased genomic instability and hyperrecombination phenotype [47], [48]. In line with a recombination-based mechanism, in *sgs1* cells SFDA was stimulated from 6 up to a factor of 20 with or without a DSB, respectively (Figure 5A, 5C). The lowest level of SFDA stimulation in the DSB system is consistent with a reduced resection and DSB repair by SSA in *sgs1* cells [49].

The percentage of colonies with a stable Ura^+^ phenotype (duplication) is increased when single-stranded oligos are used alone rather than in complementary pairs and it is reduced when a DSB is induced next to the amplicon (Figure 4E). These findings also support the fact that generation of either an extrachromosomal circle or a duplication can each be the first event in SFDA. If SFDA would always first result in formation of an extrachromosomal circle, we would expect the fraction of duplications *vs*. extrachromosomal circles to be the same in all cases (with or without the DSB, using single oligos or pairs), as the duplication would then be a secondary event, independent from the initial triggers. Duplications can also occur following integration of extrachromosomal circles in the chromosomal region containing the amplicon sequence. We found that cells initially containing extrachromosomal circles and having an unstable Ura^+^ phenotype following continuous growth in selective Ura^−^ medium became stably Ura^+^ and contained a tandem duplication of the amplicon (Figure 4D–4F). It is also possible that certain types of tandem duplications are prone to recombination and result in formation of episomes or triplications or even larger copy number forms, as the equilibrium between duplications and extrachromosomal circles could be dynamic. Application of a selective pressure that favors growth of cells with increased copy number of a specific chromosomal region is expected to enrich the culture with cells that contain more copies of the amplicon and in its most stable form. It would be very interesting to see if SFDA can be detected in mammalian cells, as there are several cases of DNA amplification resulting in tandem duplications in mammalian cells. Examples of tandem duplications include events of gene amplification observed in Chinese hamster cells [34],[50], as well as in various human cancers [51]–[53]. The study by Stephens et al., (2009) reports that the most commonly observed architecture of rearrangement in human breast cancer is tandem duplication [54]. This research also points out the fact that tandem duplications have frequently been overlooked because they are intrachromosomal and involve small chromosomal segments beyond the resolution of cytogenetics or previous generations of copy number arrays. Markedly, more recent works that exploit next generation sequencing approaches to characterize the landscape of rearrangements in ovarian and breast cancer genomes have revealed a predominance of tandem duplications [55], [56].

DNA fragments between 100–10,000 bp can be generated by DNA metabolic processes, such as DNA replication, repair and recombination (*e.g.,* cleavage products of an endonuclease during 5′-end resection or clipping of 3′-DNA tails) [57], [58], or they could form following reverse transcription of cellular RNAs into cDNAs [59]. It is of note that a recent study identified several thousands of short extrachromosomal circular DNAs (microDNAs, 200–400 bp long) in normal mouse tissue as well as in mouse and human cell lines [60]. DNA fragments could also originate from the uptake of chromosomal degradation products derived from the death and lysis of other cells [61]. While DNA degradation in necrotic cells yields mainly larger DNA fragments [62], programmed cell death, or apoptosis, produces an oligonucleosomal ladder of DNA fragments ∼180 bp [63]. Though mechanisms of extracellular DNA uptake remain mostly unknown, many studies have revealed the uptake of immunostimulatory CG-rich DNA, dsRNA, antisense oligonucleotides or simply exogenous DNA in many different kinds of cells [61], [64], [65]. DNA fragments ranging from 180 to 3,500 bp have been found in the culture medium of HeLa and HUVEC cells [62], [66], [67], and are abundant in the serum of cancer patients, as well as in people affected by autoimmune, infectious or trauma conditions [68]–[71]. Moreover, DNA derived from apoptotic cancer cells could transform healthy cells [72], [73]. We propose here that SFDA could play a significant role in gene amplification, and could therefore be a driving force for carcinogenesis. While homologous recombination compared to random integration is generally more efficient in yeast than in mammalian cells, it does occur in mammalian cells even with DNA oligos. Our recent study by Shen et al. (2011) showed that single-stranded DNA oligos are recombinogenic in human embryonic kidney (HEK-293) cells and can repair a DSB in the human genome and transfer information to chromosomal DNA in the process of DSB repair with frequencies comparable to those observed in yeast [74]. Therefore, we are confident that SFDA can potentially also occur in human cells.

In SFDA, a DSB is not required to initiate the amplification; however, the proximity of a DSB to the amplicon region increases the frequency of SFDA ∼10-fold (Figure 3B). In SFDA driven by single-stranded DNA, a DSB external to the amplicon region favors amplification initiated by the single-stranded oligo with complementarity to the non-resected 3′-end strand (Figure 3B, 3C). These data are in agreement with our previous findings that a DSB stimulates gene targeting to distant sites by single-stranded oligos in a biased manner as a consequence of 5′-end resection [39],[75],[76]. It is probably the generation of long 3′ single-stranded sequences that facilitates the annealing with the small DNA fragments and promotes SFDA especially via formation of extrachromosmal circles (only 12% duplications with 88% extrachromosomal circles with paired oligos) (Figure 4E and Figure 5B). Conversely, among the few colonies resulting from SFDA in *rad59* in the DSB system most contained duplications (79%; Figure 5D), which could reflect SSA-independent and Rad51-dependent events [29].

In summary, our results demonstrate a novel mechanism of DNA amplification driven by small DNA fragments. Our assay in yeast reveals only those events of SFDA that give rise to perfect reconstruction of the split *URA3* gene. However, we predict that many more SFDA events can be driven by short homologies, as those used in this study, or even by microhomology between the small DNA fragments and chromosomal DNA as in the FoSTeS mechanism, where microhomologies guide template switching during DNA replication [31]. It is possible that DNA fragments containing sequences of repetitive elements, such as transposons and Alu sequences [77], could more easily find homology with chromosomal regions located on the same chromosomal arm and trigger SFDA. One of the most problematic issues of gene amplification is to understanding molecular mechanisms and DNA contexts that initiate regional amplification and set the boundaries of amplicons [78]. In SFDA, small DNA fragments are the initiators of amplification and the boundaries of the amplicon region are defined by the homology tracts shared between the small DNA fragments and the target chromosomal DNA, both in the presence and in the absence of a DSB next to the amplicon. By designing DNA oligos with homology to chosen chromosomal regions, SFDA could serve as a new approach for yeast genome manipulations to generate ad hoc tandem duplications and/or extrachromosomal DNA circles. Considering that DNA fragments could be quite abundant in cells, we suggest that SFDA may be a major mechanism for initiating events of gene amplification, and that these findings may be relevant in cancer, human genetics and evolution, as well as in genome engineering.

## Materials and Methods

### Strains and plasmids used in this study

Yeast haploid strain FRO-155 (*MAT*α *his3*Δ*1 leu2*Δ*0 lys2*Δ*0 trp5*::GSHU *lys2*::Alu IR) (Table S2) contains the CORE-I-*Sce*I cassette (including the I-*Sce*I gene under the inducible *GAL1* promoter, the hygromycin resistance gene *hyg*, and the counterselectable *KIURA3* marker gene) and the I-*Sce*I site (HOT site) in *TRP5* [35]. Yeast strains KM-193 and KM-196 are two independent isolates derived from strain FRO-155 by replacing the CORE I-*Sce*I cassette with the A3-UR amplicon cassette, which derives from plasmid YRpKM1 and contains the yeast *ARS1* autonomous replicating sequence, an origin of replication (ORI) in *E. coli* cells, the ampicillin resistance gene (*Amp^R^*), and the green fluorescent protein (GFP) gene (not shown) between the split *URA3* marker gene (A3-UR) (Figure 1 and Figure 2). YRpKM1 was derived from YCp50 [79]. The *CEN4* sequence of YCp50 plasmid was deleted by digestion with *Sma*I and *Xho*I enzymes and re-ligation of Klenow-filled in overhangs. A GFP PCR product containing the *Eco*RI and *Bam*HI enzyme sites at its ends was cut by *Eco*RI and *Bam*HI enzymes and inserted into the corresponding sites of YCp50 devoid of *CEN4* to make YRpKM1. All other yeast strain backgrounds used are derived from both KM-193 and KM-196 and are described in Table S2 and Figure 1.

### Oligos used to capture SFDA events

Two oligos named AB and CD, 80 nt long, were designed to have each 40 nt of homology to either side of the A3-UR amplicon cassette (Table S1). These oligos are designed to reconstitute the split *URA3* gene of the A3-UR amplicon cassette (Figure 2). In another set of oligos, the *Sac*I or *Xba*I restriction enzyme site was introduced in the sequence of the AB and CD oligos, generating AB_S_ and CD_S_ or AB_X_ and CD_X_ oligos, respectively (Table S1 and Figure 2). To repair the DSB generated 10 kb downstream or upstream of the A3-UR amplicon cassette in strains KM-221,222 or KM-257,259 and derivatives strains, we utilized oligos e1 and f1 or R1 and R2, respectively (Table S1), as previously described [39].

### Capture of SFDA events in the absence or in the presence of an induced DSB

Yeast transformations by the AB/CD oligos or similar oligos without DSB induction in chromosomal DNA were done following the lithium acetate protocol described in Stuckey *et al*., 2011 [37] using 1 nmol of total oligos. When complementary oligos were used in a pair, the oligos were mixed together, denatured, put on ice and added to cells without any annealing *in vitro*. Cells from each oligo transformation were plated to selective Ura^−^ medium and were also diluted and plated on the rich YPD medium to determine the frequency of SFDA. Survival after yeast transformation in strains without DSB induction was 32% for WT (wild-type for genetic control genes), 34% for *rad52*Δ, 33% for *rad59*Δ, 34% for *rad51*Δ, 23% for *pol32*Δ, 28% for *ARS1*Δ, 30% for *ARS1*Δ *pol32*Δ, 7% for *sgs1*Δ, 17% for *srs2*Δ, 29% for *mph1*Δ, respectively. Yeast transformations by the AB/CD oligos or similar oligos with DSB induction to the side of the targeting chromosomal region were done as described in Storici *et al*., 2006 [39] using the DSB-repairing oligos e1 and f1 or R1 and R2 and 1 nmol of total oligos. Cells from each oligo transformation were plated to selective Ura^−^ medium and were diluted and plated on the rich YPD medium to determine the frequency of SFDA. Survival after yeast transformation in strains with DSB induction was 42% for WT, 34% for *rad52*Δ, 38% for *rad59*Δ, 34% for *rad51*Δ, 39% for *pol32*Δ, 29% for *sgs1*Δ, 47% for *srs2*Δ, 52% for *mph1*Δ, respectively.

### Ura^+^ phenotype stability assay

To determine the percentage of transformants with a stable Ura^+^ phenotype four groups of 45 colonies (total 180, unless otherwise specified) from independent transformations were picked and streaked for single colonies isolates on YPD plates and then replica plated onto Ura^−^ after two days. Growth on Ura^−^ was then scored after two days (Figure S4). The median of the four percentage values for each cell type is reported. For the stability assay coupled with genomic DNA extraction for Sothern blot hybridization, we conducted the following procedure. Cells from large or small colonies growing on Ura^−^ medium after oligo transformation were taken and used to inoculate 5 ml of liquid Ura^−^ medium. After the first day of growth (day 1), ∼500 cells were plated onto YPD to check the stability of the Ura^+^ phenotype, 50 µl of the culture were used to inoculate 5 ml of fresh Ura^−^ medium for a second day of growth, and the remaining culture was used to extract genomic DNA. After the second day of growth 50 µl of culture were used to inoculate 5 ml of fresh Ura^−^ medium for a third day of growth. This procedure was repeated until the end of the seventh day of growth, in which time ∼500 cells were plated to YPD for the stability test (Figure 4F). The reminder of the culture was used to extract genomic DNA. All comparisons of frequency values and percentages were done using the Mann-Whitney test [80].

### Standard genetic and molecular biology techniques

Standard genetics and molecular biology analyses were done as described previously ([37] and references therein). Samples for sequencing were submitted to Eurofins MWG Operon. Rescue of extrachromosomal circles from yeast cells into *E. coli* cells was performed by using the yeast plasmid preparation protocol [81].

### Identification of amplification events by Southern blot hybridization

Cells from colonies growing on Ura^−^ plates were grown in liquid Ura^−^ O/N. Genomic DNA was extracted as described [81] and digested with either the *Sac*I or *Xba*I restriction enzyme and run in a 0.6% agarose gel. Following electrophoresis and Southern blotting chromosomal regions containing the A3-UR amplicon were detected using a [γ-32P]ATP (PerkinElmer) labeled (Prime-It RmT Random Primer Labelling Kit, Agilent Technologies) 623-bp Amp^R^ specific probe. Membranes were exposed to a phosphor screen overnight. Images were taken with Typhoon Trio^+^ (GE Healthcare) and obtained with ImageQuant (GE Healthcare).

## Supporting Information

Figure S1 SFDA efficiency following transformation with AB_X_ and/or CD_X_ oligos. Presented are numbers of Ura^+^ colonies per 10^7^ viable cells obtained after transformation of yeast cells with no oligos, AB_X_ and/or CD_X_ oligos. The vertical bars correspond to the median values from six determinations; the error bars represent the range. (A) Strains used were KM-201,203. (B) Strains used were KM-221,222, in which a DSB was induced 10 kb downstream from the amplicon cassette prior to oligo transformation. (C) Strains used were KM-257,259, in which a DSB was induced 10 kb upstream of the amplicon cassette prior to oligo transformation. Frequency values obtained for the single-stranded AB_X_ and CD_X_ oligos in the different strain backgrounds were compared with each other by the Mann-Whitney test and the *p* values of the significant differences, highlighted by the asterisks, are given on top of the corresponding bars.
(PDF)

Figure S2 Restriction endonuclease digestion of extrachromosomal circles rescued from small yeast Ura^+^ colonies. Lane 1, uncut YRpKM1; lane 2, YRpKM1 cut by *Nco*I; lane 3, YRpKM1 cut by *Pst*I; lane 4, YRpKM1 cut by *Nco*I and *Bam*HI; lane 5, 2Log Ladder (New England Bio Labs); lane 6, uncut extrachromosomal circle rescued from KM-221 cells transformed by AB_X_ and CD_X_ oligos (YRpKM1_X_); lane 7, YRpKM1_X_ cut by *Xba*I; lane 8, YRpKM1_X_ cut by *Pst*I; lane 9, YRpKM1_X_ cut by *Xba*I and *Bam*HI; lane 10, 2Log Ladder; lane 11, uncut extrachromosomal circle rescued from KM-221 cells transformed by AB_S_ and CD_S_ oligos (YRpKM1_S_); lane 12, YRpKM1_S_ cut by *Sac*I; lane 13, YRpKM1_S_ cut by *Pst*I; lane 14, YRpKM1_S_ cut by *Sac*I and *Bam*HI. Size of two bands of the marker and size of detected bands from digested samples are indicated by long and short bars, respectively.
(PDF)

Figure S3 SFDA efficiency without a DSB in WT and *ARS1*Δ strains. Presented are numbers of Ura^+^ colonies per 10^7^ viable cells obtained after transformation of yeast cells with no oligos, AB_S_ and/or CD_S_ oligos. The vertical bars correspond to the median values from six determinations; the error bars represent the range. (A) Strains used were KM 201,203 and KM 347,349. (B) Percentage of colonies with stable Ura^+^ phenotype (indicative of duplication) within a random sample of 180 Ura^+^ colonies for each WT strain from the experiment shown in (A) and 60 Ura^+^ colonies from *ARS1*Δ strains.
(PDF)

Figure S4 Example of Ura^+^ stability test. The result for the stability test for 6 random colonies is shown. The 6 streaks on YPD show growth. After replica plating on Ura^−^ media, only two of the six streaks are growing. Yeast cells were determined to have a stable Ura^+^ phenotype if all colonies transferred from the YPD to the Ura^−^ media grew on the Ura^−^ plates. Differently, when we observed poor growth on the Ura^−^ media, yeast cells were determined to have an unstable Ura^+^ phenotype.
(TIF)

Table S1 Oligos used in this study. The structures of the oligos used in this study are described from the 5′ end, with restriction endonuclease site sequences shown in bold and underlined. The presence of the *Sac*I site within the AB_S_ and CD_S_ oligo sequence changes the 69^th^ serine codon (TCC) of *URA3* and the 70^th^ methionine codon (ATG) to glutamine (GAG) and leucine (CTC), respectively. The presence of the *Xba*I site within the AB_X_ and CD_X_ oligo sequence creates a silent mutation on codon 69^th^ serine of *URA3* (TCC to TCT) and changes the 70^th^ codon methionine to arginine codon (ATG to AGA). These changes into the *URA3* coding sequence do not alter the functionality of the Ura3 protein.
(PDF)

Table S2 Yeast strains used in this study. ^a^GSHU *lys2::*AluIR contains the GSHU cassette (including the I-*Sce*I gene under the inducible *GAL1* promoter, the hygromycin resistance gene *hyg*, and the counterselectable marker gene *KIURA3*) and the I-*Sce*I site (HOT site) in *TRP5.* ^b^A3-UR amplicon cassette derivative from YRpKM1 plasmid containing an autonomous replicating sequence (*ARS1*), bacterial origin of replication (ORI), ampicillin resistance gene (*Amp^R^*), the green fluoresence protein (GFP) gene and the split *URA3* gene (A3-UR). ^c^ *LEU2* is integrated downstream of UR. ^d^GSHdw cassette (including the I-*Sce*I gene under the inducible *GAL1* promoter and *hyg*) and the I-*Sce*I (HOT) site are integrated 10 kb downstream of the UR sequence. ^e^GSHup cassette (including the I-*Sce*I gene under the inducible *GAL1* promoter and *hyg*) and the I-*Sce*I (HOT) site are integrated 10 kb upstream of the A3 sequence. ^f^Single deletion strains contain the *kanMX4* module in place of the chosen gene, and are all derivatives of KM-201,203 or KM-221,222 strains.
(PDF)

Table S3 SFDA driven by oligos with homology as short as 20 bases. Mean of Ura^+^ colonies per 10^7^ viable cells obtained after transformation of wild-type and *sgs1* mutant cells with no oligos, A20B60_S_, C60D20_S_ or A20B60_S_+C60D20_S_ oligos from six determinations; the range is shown in parenthesis.
(PDF)
