# Supplementary figures and images for: A Mechanism of Gene Amplification Driven by Small DNA Fragments
Source: PLoS Genet. 2012 Dec 13;8(12):e1003119. doi: 10.1371/journal.pgen.1003119 (PMC3521702; doi:10.1371/journal.pgen.1003119)

Figure S1

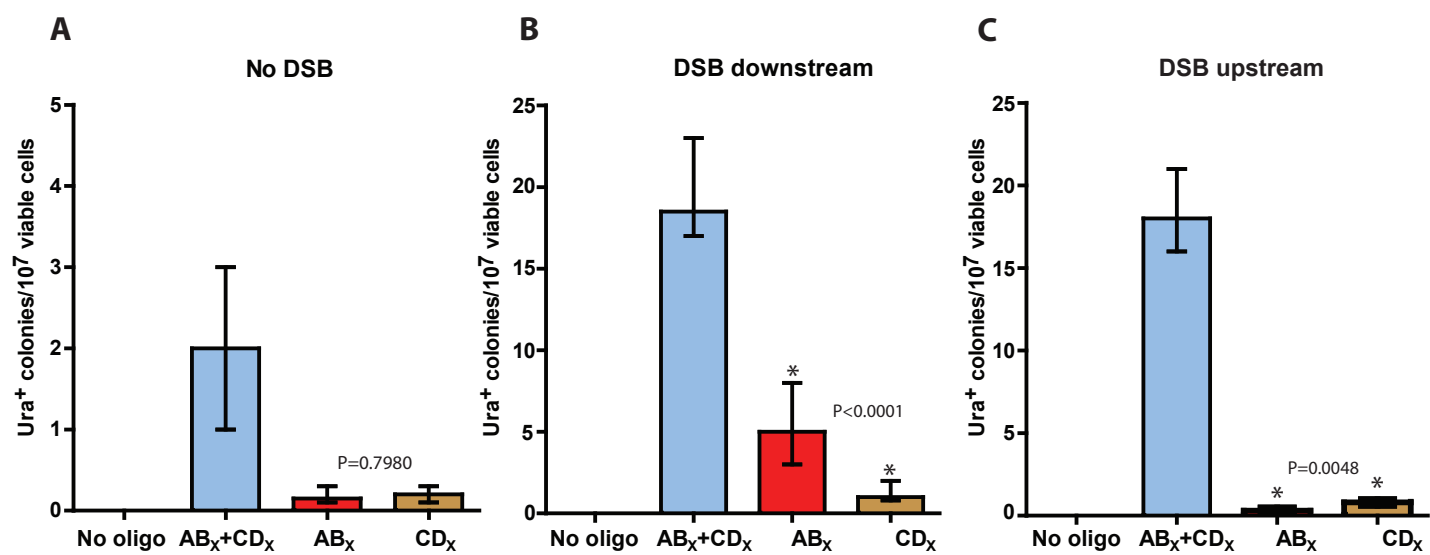

Supplement: Figure S1 — SFDA efficiency following transformation with ABX and/or CDX oligos. Presented are numbers of Ura+ colonies per 107 viable cells obtained after transformation of yeast cells with no oligos, ABX and/or CDX oligos. The vertical bars correspond to the median values from six determinations; the error bars represent the range. (A) Strains used were KM-201,203. (B) Strains used were KM-221,222, in which a DSB was induced 10 kb downstream from the amplicon cassette prior to oligo transformation. (C) Strains used were KM-257,259, in which a DSB was induced 10 kb upstream of the amplicon cassette prior to oligo transformation. Frequency values obtained for the single-stranded ABX and CDX oligos in the different strain backgrounds were compared with each other by the Mann-Whitney test and the p values of the significant differences, highlighted by the asterisks, are given on top of the corresponding bars. (PDF) [file pgen.1003119.s001.pdf]

Figure S2

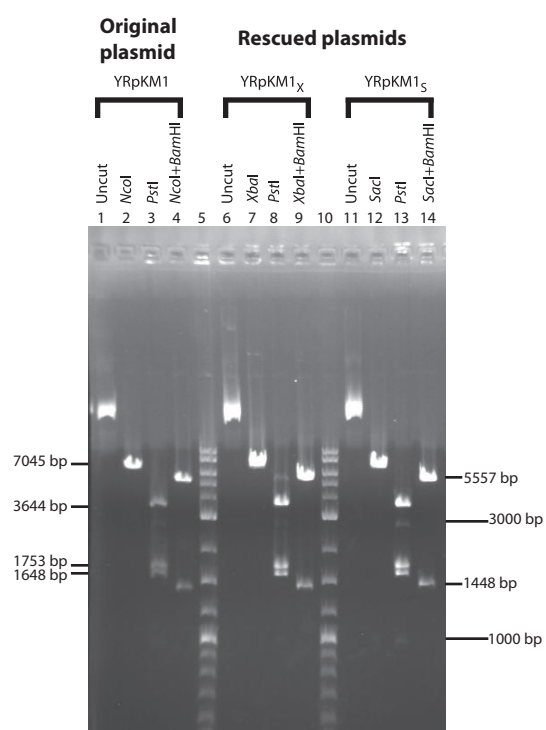

Supplement: Figure S2 — Restriction endonuclease digestion of extrachromosomal circles rescued from small yeast Ura+ colonies. Lane 1, uncut YRpKM1; lane 2, YRpKM1 cut by NcoI; lane 3, YRpKM1 cut by PstI; lane 4, YRpKM1 cut by NcoI and BamHI; lane 5, 2Log Ladder (New England Bio Labs); lane 6, uncut extrachromosomal circle rescued from KM-221 cells transformed by ABX and CDX oligos (YRpKM1X); lane 7, YRpKM1X cut by XbaI; lane 8, YRpKM1X cut by PstI; lane 9, YRpKM1X cut by XbaI and BamHI; lane 10, 2Log Ladder; lane 11, uncut extrachromosomal circle rescued from KM-221 cells transformed by ABS and CDS oligos (YRpKM1S); lane 12, YRpKM1S cut by SacI; lane 13, YRpKM1S cut by PstI; lane 14, YRpKM1S cut by SacI and BamHI. Size of two bands of the marker and size of detected bands from digested samples are indicated by long and short bars, respectively. (PDF) [file pgen.1003119.s002.pdf]

Figure S3

A

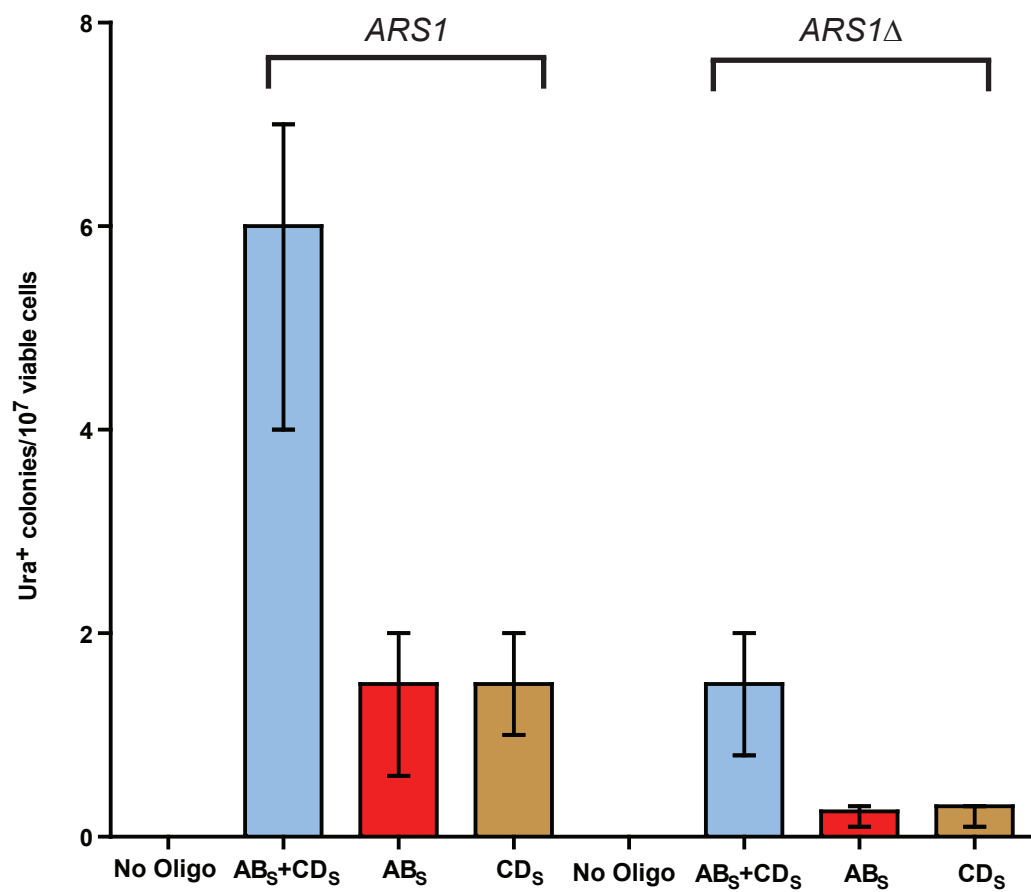

B

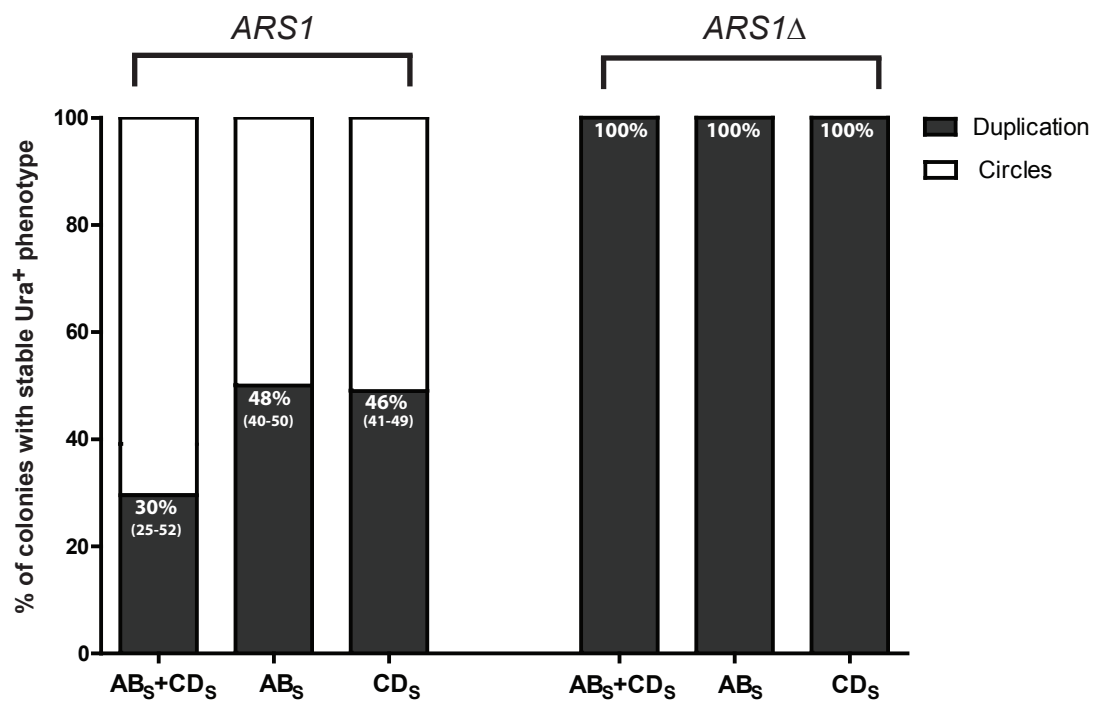

Supplement: Figure S3 — SFDA efficiency without a DSB in WT and ARS1Δ strains. Presented are numbers of Ura+ colonies per 107 viable cells obtained after transformation of yeast cells with no oligos, ABS and/or CDS oligos. The vertical bars correspond to the median values from six determinations; the error bars represent the range. (A) Strains used were KM 201,203 and KM 347,349. (B) Percentage of colonies with stable Ura+ phenotype (indicative of duplication) within a random sample of 180 Ura+ colonies for each WT strain from the experiment shown in (A) and 60 Ura+ colonies from ARS1Δ strains. (PDF) [file pgen.1003119.s003.pdf]

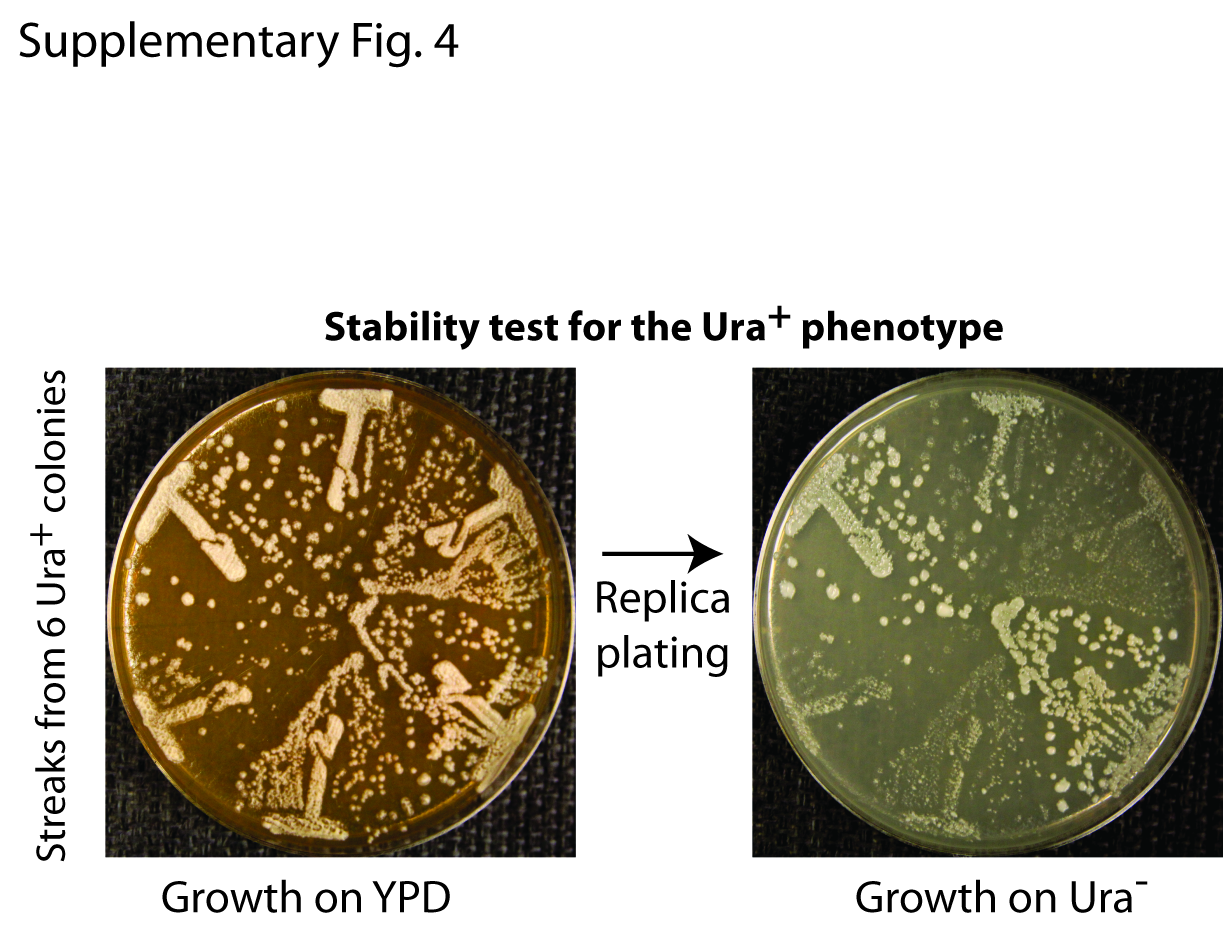

Supplement: Figure S4 — Example of Ura+ stability test. The result for the stability test for 6 random colonies is shown. The 6 streaks on YPD show growth. After replica plating on Ura− media, only two of the six streaks are growing. Yeast cells were determined to have a stable Ura+ phenotype if all colonies transferred from the YPD to the Ura− media grew on the Ura− plates. Differently, when we observed poor growth on the Ura− media, yeast cells were determined to have an unstable Ura+ phenotype. (TIF) [file pgen.1003119.s004.tif]
