# Supplementary material for: A Mechanism of Gene Amplification Driven by Small DNA Fragments
Source: PLoS Genet. 2012 Dec 13;8(12):e1003119. doi: 10.1371/journal.pgen.1003119 (PMC3521702; doi:10.1371/journal.pgen.1003119)
Supplement: Table S1 — Oligos used in this study. The structures of the oligos used in this study are described from the 5′ end, with restriction endonuclease site sequences shown in bold and underlined. The presence of the SacI site within the ABS and CDS oligo sequence changes the 69th serine codon (TCC) of URA3 and the 70th methionine codon (ATG) to glutamine (GAG) and leucine (CTC), respectively. The presence of the XbaI site within the ABX and CDX oligo sequence creates a silent mutation on codon 69th serine of URA3 (TCC to TCT) and changes the 70th codon methionine to arginine codon (ATG to AGA). These changes into the URA3 coding sequence do not alter the functionality of the Ura3 protein. (PDF) [file pgen.1003119.s005.pdf]

Table S1. Oligos used in this study.

Name	Site	Size	Sequence
AB	*Nco*I	80	5'GGCGGATAATGCCTTTAGCGGCTTAACTGTGCCCT**CCATGG**AAAAATCAGTCAAGATATCCACATGTGTTTTTAGTAAAC
CD	*Nco*I	80	5'GTTTACTAAAAACACATGTGGATATCTTGACTGATTTTT**CCATGG**AGGGCACAGTTAAGCCGCTAAAGGCATTATCCGCC
AB_S	*Sac*I	80	5'GGCGGATAATGCCTTTAGCGGCTTAACTGTGCCCTC**GAGCTC**AAAATCAGTCAAGATATCCACATGTGTTTTTAGTAAAC
CD_S	*Sac*I	80	5'GTTTACTAAAAACACATGTGGATATCTTGACTGATTTT**GAGCTC**GAGGGCACAGTTAAGCCGCTAAAGGCATTATCCGCC
AB_X	*Xba*I	80	5'GGCGGATAATGCCTTTAGCGGCTTAACTGTGCCCTC**TCTAGA**AAAATCAGTCAAGATATCCACATGTGTTTTTAGTAAAC
CD_X	*Xba*I	80	5'GTTTACTAAAAACACATGTGGATATCTTGACTGATTTT**TCTAGA**GAGGGCACAGTTAAGCCGCTAAAGGCATTATCCGCC
$A20B60_S$	*Sac*I	80	5'AAGAGTAAAAAATTGTACTTGGCGGATAATGCCTTTAGCGGCTTAACTGTGCCCTC**GAGCTC**AAAATCAGTCAAGATATC
$C60D20_S$	*Sac*I	80	5'GATATCTTGACTGATTTT**GAGCTC**GAGGGCACAGTTAAGCCGCTAAAGGCATTATCCGCCAAGTACAATTTTTTACTCTT
e1		80	5'TCCGAAGCTGGCCAATTGATACAATTAATTGACATCAGCAGACAGCAAATGCACTTGATATACGCAGCTTAAAGCAAGGG
f1		80	5'CCCTTGCTTTAAGCTGCGTATATCAAGTGCATTTGCTGTCTGCTGATGTCAATTAATTGTATCAATTGGCCAGCTTCGGA
R1		80	5'GTTGTATATACCACTACTCTGTGATTTTTTTTCACTCTTGGGAAGGAAAGAAAAAGTGAAAACATCAACTGGTAAGTGTC
R2		80	5'GACACTTACCAGTTGATGTTTTCACTTTTTCTTTCCTTCCCAAGAGTGAAAAAAAATCACAGAGTAGTGGTATATACAAC
Trp5.A3-F		80	5'GTCTAAGAGAGTTGGAAAAGGGTTTTGATGAAGCTGTCGCCATGGAGGGCACAGTTAAGCCGCTAAAGGCATTATCCGCC
Trp5.A3-R		80	5'GGCGGATAATGCCTTTAGCGGCTTAACTGTGCCCTCCATGGCGACAGCTTCATCAAAACCCTTTTCCAACTCTCTTAGAC
Trp5.UR-F		80	5'TGTTTACTAAAAACACATGTGGATATCTTGACTGATTTTTCCGATCCCACATTCTGGGAAGACTTCAAATCCTTGTATTCT
Trp5.UR-R		80	5'AGAATACAAGGATTTGAAGTCTTCCCAGAATGTGGGATCGGAAAAATCAGTCAAGATATCCACATGTGTTTTTAGTAAACA

The structures of the oligos used in this study are described from the 5′ end, with restriction endonuclease site sequences shown in bold and underlined. The presence of the *Sac*I site within the AB_S and CD_S oligo sequence changes the 69th serine codon (TCC) of *URA3* and the 70th methionine codon (ATG) to glutamine (GAG) and leucine (CTC), respectively. The presence of the *Xba*I site within the AB_X and CD_X oligo sequence creates a silent mutation on codon 69th serine of *URA3* (TCC to TCT) and changes the 70th codon methionine to arginine codon (ATG to AGA). These changes into the *URA3* coding sequence do not alter the functionality of the Ura3 protein.
