# Supplementary material for: A Mechanism of Gene Amplification Driven by Small DNA Fragments
Source: PLoS Genet. 2012 Dec 13;8(12):e1003119. doi: 10.1371/journal.pgen.1003119 (PMC3521702; doi:10.1371/journal.pgen.1003119)
Supplement: Table S2 — Yeast strains used in this study. aGSHU lys2::AluIR contains the GSHU cassette (including the I-SceI gene under the inducible GAL1 promoter, the hygromycin resistance gene hyg, and the counterselectable marker gene KIURA3) and the I-SceI site (HOT site) in TRP5. bA3-UR amplicon cassette derivative from YRpKM1 plasmid containing an autonomous replicating sequence (ARS1), bacterial origin of replication (ORI), ampicillin resistance gene (AmpR), the green fluoresence protein (GFP) gene and the split URA3 gene (A3-UR). c LEU2 is integrated downstream of UR. dGSHdw cassette (including the I-SceI gene under the inducible GAL1 promoter and hyg) and the I-SceI (HOT) site are integrated 10 kb downstream of the UR sequence. eGSHup cassette (including the I-SceI gene under the inducible GAL1 promoter and hyg) and the I-SceI (HOT) site are integrated 10 kb upstream of the A3 sequence. fSingle deletion strains contain the kanMX4 module in place of the chosen gene, and are all derivatives of KM-201,203 or KM-221,222 strains. (PDF) [file pgen.1003119.s006.pdf]

Table S2. Yeast strains used in this study.

Name	Relevant genotype	Source
FRO-155	*MATα his3Δ1 leu2Δ0 lys2Δ0 trp5::*GSHU *lys2::*AluIR[a]	Storici et al., 2003
KM-193	FRO-155, GSHU*::*A3-UR[b]	This work
KM-196	FRO-155, GSHU*::*A3-UR	This work
KM-201	KM-193, UR-*LEU2*[c]	This work
KM-203	KM-196, UR-*LEU2*	This work
KM-209	KM-193, UR*::LEU2*	This work
KM-211	KM-193, UR*::LEU2*	This work
KM-221	KM-201, GSHdw[d]	This work
KM-222	KM-203, GSHdw	This work
KM-257	KM-201, GSHup[e]	This work
KM-259	KM-203, GSHup	This work
KM-261	KM-221, *rad52Δ::kanMX4*[f]	This work
KM-263	KM-222, *rad52Δ::kanMX4*	This work
KM-265	KM-201, *rad52Δ::kanMX4*	This work
KM-267	KM-203, *rad52Δ::kanMX4*	This work
KM-269	KM-221, *rad51Δ::kanMX4*	This work
KM-271	KM-222, *rad51Δ::kanMX4*	This work
KM-273	KM-201, *rad51Δ::kanMX4*	This work
KM-275	KM-203, *rad51Δ::kanMX4*	This work
KM-277	KM-221, *pol32Δ::kanMX4*	This work
KM-279	KM-222, *pol32Δ::kanMX4*	This work
KM-281	KM-201, *pol32Δ::kanMX4*	This work
KM-283	KM-203, *pol32Δ::kanMX4*	This work
KM-315	KM-201, *rad59Δ::kanMX4*	This work
KM-317	KM-203, *rad59Δ::kanMX4*	This work
KM-319	KM-221, *rad59Δ::kanMX4*	This work
KM-321	KM-222, *rad59Δ::kanMX4*	This work
KM-323	KM-201, *mph1Δ::kanMX4*	This work
KM-325	KM-203, *mph1Δ::kanMX4*	This work
KM-327	KM-221, *mph1Δ::kanMX4*	This work
KM-329	KM-222, *mph1Δ::kanMX4*	This work
KM-331	KM-201, *srs2Δ::kanMX4*	This work
KM-333	KM-203, *srs2Δ::kanMX4*	This work
KM-335	KM-221, *srs2Δ::kanMX4*	This work
KM-337	KM-222, *srs2Δ::kanMX4*	This work
KM-339	KM-201, *sgs1Δ::kanMX4*	This work
KM-341	KM-203, *sgs1Δ::kanMX4*	This work
KM-343	KM-221, *sgs1Δ::kanMX4*	This work

KM-345	KM-222, *sgs1Δ::kanMX4*	This work
KM-347	KM-201, *ARS1Δ::kanMX4*	This work
KM-349	KM-203, *ARS1Δ::kanMX4*	This work
KM-379	KM-201, *rad1Δ::kanMX4*	This work
KM-381	KM-203, *rad1Δ::kanMX4*	This work
KM-383	KM-201, *rad10Δ::kanMX4*	This work
KM-385	KM-203, *rad10Δ::kanMX4*	This work
KM-387	KM-221, *rad1Δ::kanMX4*	This work
KM-389	KM-222, *rad1Δ::kanMX4*	This work
KM-391	KM-221, *rad10Δ::kanMX4*	This work
KM-393	KM-222, *rad10Δ::kanMX4*	This work

[a]GSHU *lys2::*AluIR contains the GSHU cassette (including the I-*Sce*I gene under the inducible *GAL1* promoter, the hygromycin resistance gene *hyg*, and the counterselectable marker gene *KlURA3*) and the I-*Sce*I site (HOT site) in *TRP5*.

[b]A3-UR amplicon cassette derivative from YRpKM1 plasmid containing an autonomous replicating sequence (*ARS1*), bacterial origin of replication (ORI), ampicillin resistance gene (Amp^R), the green fluoresence protein (GFP) gene and the split *URA3* gene (A3-UR).

[c]*LEU2* is integrated downstream of UR.

[d]GSHdw cassette (including the I-*Sce*I gene under the inducible *GAL1* promoter and *hyg*) and the I-*Sce*I (HOT) site are integrated 10 kb downstream of the UR sequence.

[e]GSHup cassette (including the I-*Sce*I gene under the inducible *GAL1* promoter and *hyg*) and the I-*Sce*I (HOT) site are integrated 10 kb upstream of the A3 sequence.

[f]Single deletion strains contain the *kanMX4* module in place of the chosen gene, and are all derivatives of KM-201,203 or KM-221,222 strains.
