# Supplementary material for: A Mechanism of Gene Amplification Driven by Small DNA Fragments
Source: PLoS Genet. 2012 Dec 13;8(12):e1003119. doi: 10.1371/journal.pgen.1003119 (PMC3521702; doi:10.1371/journal.pgen.1003119)
Supplement: Table S3 — SFDA driven by oligos with homology as short as 20 bases. Mean of Ura+ colonies per 107 viable cells obtained after transformation of wild-type and sgs1 mutant cells with no oligos, A20B60S, C60D20S or A20B60S+C60D20S oligos from six determinations; the range is shown in parenthesis. (PDF) [file pgen.1003119.s007.pdf]

Table S3. SFDA driven by oligos with homology as short as 20 bases.

Strain	No oligo	$A20B60_S$	$C60D20_S$	$A20B60_S$+$C60D20_S$
No DSB				
KM-201,203 (WT)	<0.01 (0-0)	0.06 (0-0.2)	0.06 (0-0.2)	0.06 (0-0.2)
KM-339,341 (Δ*sgs1*)	<0.01 (0-0)	0.1 (0-0.4)	0.1 (0-0.4)	0.3 (0-1)
DSB				
KM-221,222 (WT)	<0.01 (0-0)	0.06 (0-0.2)	<0.01 (0-0)	0.05 (0-0.1)
KM-343,345 (Δ*sgs1*)	<0.01 (0-0)	0.1 (0-0.3)	<0.01 (0-0)	1 (0.5-2)

Mean of Ura^+ colonies per 10^7 viable cells obtained after transformation of wild-type and *sgs1* mutant cells with no oligos, $A20B60_S$, $C60D20_S$ or $A20B60_S$ + $C60D20_S$ oligos from six determinations; the range is shown in parenthesis.
